# Psychometric Analysis of Items Evaluating Health Belief Model Constructs in Social Media Posts: Application of Rasch Measurement Model

**DOI:** 10.3390/bs15020204

**Published:** 2025-02-13

**Authors:** Xiaofeng Jia, Soyeon Ahn

**Affiliations:** 1 School of Media & Communication, Bowling Green State University, Bowling Green, OH 43403, USA; 2 Department of Educational and Psychological Studies, School of Education and Human Development, University of Miami, Coral Gables, FL 33146, USA

**Keywords:** Rasch measurement model, social media messages, health promotion, psychometric analysis, health belief model

## Abstract

Social media is a crucial tool for health communication as it provides an immediate, wide-reaching platform to share information, correct misinformation, and promote health behaviors. The Health Belief Model (HBM) offers a structured approach for designing more effective social media messages by employing unique constructs predicting health behaviors, such as severity, susceptibility, benefits, barriers, and self-efficacy. While prior research has explored HBM constructs in health messages, most studies have collected the survey data with items lacking robust psychometric evidence, particularly in evaluating social media posts. This study addresses this gap by using Rasch Measurement Theory (RMT) to analyze the psychometric properties of HBM items evaluating social media posts promoting COVID-19 vaccination. The findings indicate that severity, benefits, and barriers are the most reliable HBM constructs in social media posts, while susceptibility and self-efficacy are underutilized in health messaging for social media. Also, dimensionality analysis confirms distinct patterns, but unexplained variance suggests that additional factors influence vaccine messaging, raising validity concerns. These results underscore the need to refine HBM-based message strategies by emphasizing overlooked constructs and improving item effectiveness. This study provides guidelines for using HBM-related measures in social media by establishing comprehensive psychometric properties, especially when applied in social media contexts. It also presents practical guidelines for designing and evaluating social media health messages, ensuring they effectively utilize HBM constructs to promote positive health behaviors. Future research should explore measurement invariance and content creators’ emphasis on HBM constructs, leveraging high-engagement tweets while expanding to diverse perspectives for broader applicability.

## 1. Introduction

Social media platforms have become a vital conduit for disseminating health information to a global audience (Lapointe et al., 2014). Governments, health organizations, and individuals have turned to platforms like Twitter and Facebook to share important policy updates, correct misinformation, and promote health behavior efforts. The vast reach and immediacy of social media make it an indispensable tool for health communication, especially in times of a global health crisis (Kothari et al., 2022). However, designing effective health messages requires a deeper understanding of how various message components influence public engagement and subsequent behavior-related outcomes.

One well-established framework for designing and evaluating health messages is the Health Belief Model (HBM), which predicts health behaviors based on five key constructs: severity, susceptibility, benefits, barriers, and self-efficacy (Rosenstock, 1974). Previous survey-based studies have shown an acceptable intercoder reliability of HBM measures (Bateman et al., 2021; Chen et al., 2021). However, there is a lack of studies supporting the psychometric evidence of HBM measures, which are important for ensuring their reliability and validity in evaluating social media messages.

Prior research has shown that certain HBM constructs are frequently emphasized in social media health messages. For instance, government agencies promoting COVID-19 prevention behaviors often highlight the benefits of vaccination (French et al., 2020). However, simply detecting the presence of these constructs in social media posts is not enough to understand their effectiveness in crafting impactful messages. Further research is needed to establish the psychometric properties of social media posts in their use of HBM constructs.

This study addresses this gap by applying Rasch Measurement Theory (RMT) to assess the psychometric properties of HBM-based items used to evaluate social media posts promoting COVID-19 vaccination. The Rasch model provides a rigorous statistical approach for determining the reliability and validity of measurement items (Kleppang et al., 2020). Reliability refers to the consistency of a measure, while validity concerns whether a measure accurately captures the concept (Roberts & Priest, 2006). By analyzing the performance of items related to severity, susceptibility, benefits, barriers, and self-efficacy, this research aims to refine HBM-based assessments of social media messages and ensure that they effectively capture key health behavior constructs.

The implications of this research are substantial. Theoretically, it enhances our understanding of HBM measures by applying them to social media content, highlighting the strengths and limitations of each construct. This study offers guidelines for the future use of HBM-related measures in social media by establishing comprehensive psychometric properties, particularly when used in social media contexts. Practically, the findings offer valuable insights for public health officials and social media content creators by identifying underutilized or inconsistently communicated HBM constructs. This research can guide the development of more effective health messages that leverage the full potential of the HBM. Ultimately, this study aims to improve the effectiveness of social media in promoting health behaviors, contributing to better public health outcomes.

The present study is focused on two primary research questions:

RQ1: How reliable are the HBM items in assessing whether health-related social media posts represent HBM constructs?
RQ2: How valid are the HBM items in evaluating whether health-related social media posts represent HBM constructs?

## 2. Literature Review

### 2.1. Social Media in Health Promotion

Social media is a dynamic and rapidly evolving resource with local, regional, and global reach, catering to diverse demographic groups (Bruns, 2015; Kaplan & Haenlein, 2010). These platforms provide opportunities to connect with friends, family, and individuals with similar interests, and to access a wide range of information and perspectives on numerous social topics and issues (Arceneaux & Dinu, 2018; Sawyer & Chen, 2012; Smith et al., 2011).

The potential of social media to positively influence health attitudes and behaviors is substantial (Centola, 2013). Numerous studies highlight this impact. For instance, the results of a meta-analysis containing 17 studies with a cumulative sample size of 3561 showed that social media interventions had a significant moderate-sized effect on behavior change among populations with health disparities (Vereen et al., 2023). Similarly, a systematic review containing 99 studies identified a model of social media effects in public health communication campaigns. The results suggested that campaign exposure can lead to individual behavior change and improved health outcomes, either through a direct or indirect pathway (Kite et al., 2023).

Utilizing appropriate messaging strategies in social media posts is important for promoting health behaviors (Gough et al., 2017; Huang et al., 2023; Liu et al., 2025). Research highlights that effective messaging strategies in social media posts can substantially influence user engagement and health behaviors (Friedman et al., 2022; Jia et al., 2024). Tailoring messages to specific audiences is essential, as it increases relevance and engagement, leading to higher rates of message acceptance and behavior change (Mao et al., 2024; Rimer & Kreuter, 2006). For instance, communicating the advantages of adopting healthy behaviors, such as improved well-being and disease prevention, can motivate individuals to take action (Naslund et al., 2020). Conversely, recognizing and mitigating perceived barriers, such as cost, time, or inconvenience, can reduce resistance and facilitate behavior change (Antheunis et al., 2013). To navigate these challenges, a thorough understanding of how social media messaging strategies influence health behaviors is crucial.

### 2.2. Health Belief Model

The Health Belief Model (HBM) is a widely used framework for understanding health behaviors across various contexts and populations (Rosenstock, 1974). It is popular in developing interventions aimed at promoting healthy behaviors, such as vaccination and disease prevention (Jones et al., 2014). The HBM posits that health-related behavior is influenced by an individual’s perception of the seriousness and likelihood of a health threat, as well as the perceived benefits and barriers to adopting a recommended health action (Rosenstock, 1974).

The HBM consists of five key components: perceived susceptibility, perceived severity, perceived benefits, perceived barriers, and self-efficacy. It focuses on individuals’ perceptions of a threat and their evaluation of suggested behaviors (Janz & Becker, 1984). Threat perception includes beliefs about one’s susceptibility to a health issue and the severity of its consequences. The evaluation of a recommended behavior involves beliefs about its benefits and the obstacles to adopting it. Self-efficacy refers to the confidence in one’s ability to perform the behavior, such as getting vaccinated (Rosenstock, 1974).

Numerous reviews have demonstrated the HBM’s effectiveness in predicting and explaining preventive health behaviors. Perceived benefits and barriers are consistently strong predictors, while perceived susceptibility and severity often show weaker associations with behavior change (Carpenter, 2010). Recent studies, particularly on COVID-19 vaccination, have reaffirmed the HBM’s predictive power. Perceived susceptibility, severity, benefits, barriers, cues to action, and self-efficacy have all been identified as predictors of COVID-19 vaccination willingness and behaviors (Bateman et al., 2021; Chen et al., 2021; Mercadante & Law, 2021).

### 2.3. Development and Application of Health Belief Model Measures

The HBM constructs have been operationalized through validated scales and questionnaires, enabling researchers to quantitatively evaluate each dimension of health beliefs (Champion & Skinner, 2008; Janz & Becker, 1984). These measures have been extensively utilized in previous research to predict and explain a wide range of health behaviors. For example, Carpenter (2010) conducted a meta-analysis that confirmed the reliability and predictive validity of HBM measures across different populations and health contexts.

Recent studies have employed these measures to evaluate health-related content on social media. By analyzing the application of Health Belief Model (HBM) constructs—such as susceptibility, severity, benefits, and barriers—in social media messages, researchers can gain insights into how and to what extent social media content creators utilize these constructs to promote health behaviors. For example, HBM items have been applied to analyze content related to the HPV vaccine (Li & Zheng, 2020) and COVID-19 vaccine (Jia et al., 2024; Raamkumar et al., 2020) on social media. These applications need further investigation into the reliability and validity of HBM items to be utilized in various contexts. Establishing robust metrics will ensure that the insights derived from social media evaluations accurately reflect the constructs of the HBM and contribute to effective health promotion strategies.

### 2.4. Rasch Measurement Model

Rasch Measurement Theory (RMT) is a psychometric model used to analyze and measure data from assessments (Rasch, 1993). The core principle of RMT is to create a linear, additive scale that measures a latent trait by evaluating the probability of a particular response to an item based on the trait level of the respondent and the difficulty of the item.

The Rasch model provides a robust alternative to Classical Test Theory (CTT) for evaluating the validity, reliability, and fairness of study quality ratings (Engelhard, 2013; Wang et al., 2022). While CTT often encounters challenges such as calibrating the item difficulty and estimating the measurement error, as well as the sample dependence of coefficient measures, the Rasch model offers several advantages:

(1) Dimensionality Assessment: it allows for the evaluation of the assessment’s dimensionality, ensuring that the measure accurately reflects the construct being assessed.
(2) Item Analysis: by examining item-fit statistics, the model identifies items that may be redundant or that measure different constructs or irrelevant factors.
(3) Difficulty Level Identification: the model flags items based on their difficulty levels, which helps in refining the assessment.
(4) Response Category Evaluation: it assesses whether the response categories effectively distinguish items based on their quality.

Rasch Measurement Theory (RMT) is particularly useful for analyzing categorical data, such as dummy variables, in relation to the ability of the person (e.g., rater or reviewer) and the difficulty of the items (Kleppang et al., 2020). RMT leverages information from both the person and the item to estimate the probability that a person with a given ability level will respond correctly to an item. This probabilistic framework ensures that RMT is falsifiable and adheres to the linearity assumptions required for parametric statistical tests. Fit statistics for both person fit and item fit provide evidence of validity, demonstrating how well the model predicts responses.

Andrich’s (1982) development of the Rasch Rating Scale Model (RSM), also known as the polytomous Rasch model, extends this approach to data with more than two ordinal categories. This model estimates person locations on a continuous latent variable, item difficulties, and a set of thresholds fixed across items. Additionally, RMT transforms ordinal data into logits, facilitating the appropriate use of parametric statistical analysis without the risk of Type I and II error inflation. The item parameters estimated by RMT are generally invariant to the population from which the estimates are derived, allowing for greater generalization and more sophisticated applications.

Recent applications of RMT extend beyond traditional psychometric assessments, offering novel insights into the evaluation of measurement constructs in diverse contexts, including social media research. Previous research uses Classical Test Theory (CTT) and other approaches that rely on sample-dependent metrics and assume that all items contribute equally to a construct (Huynh et al., 2021; Jia et al., 2023). RMT provides item-level diagnostics that enhance the precision and generalizability of measurement models. In this study, the Many-Facet Rasch Model (MFRM) enables a refined evaluation of HBM constructs in social media posts by identifying potential measurement biases, ensuring dimensional validity, and improving scale functioning.

### 2.5. Psychometric Properties in Rasch Measurement Model

Rasch Measurement Theory (RMT) is widely used to establish validity, reliability, and fairness in measurement (American Educational Research Association et al., 2014). This study focuses on reliability and validity, which are essential for evaluating the psychometric properties of HBM-based measures in social media message analysis.

Reliability refers to the consistency of scores across different evaluations (Ebel, 1951). While traditional methods like the coefficient alpha from Classical Test Theory (CTT) assess internal consistency, they assume interval-level data, which may misrepresent ordinal survey responses (Linacre, 1991). RMT overcomes this limitation by using the reliability of separation index, which quantifies how distinctly the latent measures are spread along the scale (Andrich, 1982). This index ranges from 0 to 1, with higher values indicating greater measurement precision.

Mathematically, the reliability of separation is defined as

$$Reliability=\frac{{SD}^{2}-MSE}{{SD}^{2}}$$

*SD* represents the standard deviation of Rasch measures for a specific facet (such as students, tasks, or raters), and *MSE* is the average Mean Squared Error of these measures. Higher reliability values are preferred as they indicate a more accurate and reliable measurement process, effectively capturing the distinctions among the latent measures across the continuum.

Validity concerns whether a measurement instrument accurately captures the intended construct (American Educational Research Association et al., 2014). RMT assesses validity through Infit and Outfit Mean Square (MnSq) statistics, which evaluate how well individual items align with the latent trait being measured. The Infit MnSq statistic detects irregular response patterns, while the Outfit MnSq statistic identifies extreme deviations. An expected MnSq value of 1.0 indicates a good fit, while significant deviations suggest potential measurement issues (Ahn et al., 2023). These fit statistics help to identify misfitting items, strengthening the internal validity of the instrument. Table 1 shows the interpretation of the Infit and Outfit MnSq values (Ahn et al., 2023).

## 3. Method

### 3.1. Sample

A sample of tweets was generated from the CoVaxxy dataset, which comprises public English-language tweets about COVID-19 vaccines collected via Twitter’s Application Programming Interface (API) from 4 January 2021 to 20 January 2022 (DeVerna et al., 2021). Given the extensive size of the CoVaxxy dataset (*n* = 308,577,272 tweets), a purposive sample was extracted based on specific criteria. To ensure relevance to major COVID-19 vaccine events, tweets were selected from dates on which substantial vaccine-related events occurred, such as the Centers for Disease Control and Prevention (CDC) recommending a pause on the Johnson & Johnson (J&J) vaccine. Next, the study focused on the characteristics of original tweets and their creators to understand user engagement; therefore, only original tweets were included, and retweets were excluded. Next, the sample prioritized tweets with high user engagement, selecting the top 1000 tweets with the most retweets and the top 1000 tweets with the most favorites. Lastly, to eliminate bot-generated content, Botometer was employed to assign a Complete Automation Probability (CAP) score to each Twitter account, and accounts with a CAP score above 95% were identified as bots and excluded.

Applying these criteria to the CoVaxxy dataset resulted in a final compilation of 1210 tweets authored by 699 distinct users. These tweets were then coded by two coders to identify the use of HBM constructs, revealing that 449 out of the 1210 tweets were related to the COVID-19 vaccine and contained HBM constructs.

### 3.2. Coding Scheme

Each tweet was coded as a categorical variable to measure each HBM construct, using 1 for presence and 0 for absence. The coding scheme was adapted from HBM measures used in COVID-19 vaccine research (Al-Metwali et al., 2021; Chen et al., 2021; Wong et al., 2021; Yu et al., 2021). The coding units included five items for perceived susceptibility (e.g., high death rate, potential fatality of COVID-19), five items for perceived severity (e.g., impact on children, elderly, pregnant women), six items for perceived benefits of vaccination (e.g., reduced infection risk, alleviation of anxiety), seven items for perceived barriers to vaccination (e.g., side effects, inconvenience), and three items for perceived self-efficacy (e.g., availability of free vaccines, ease of access).

To ensure the relevance of these items for evaluating tweets about the COVID-19 vaccine, we randomly selected 1000 tweets from the “CoVaxxy” dataset, in addition to our existing dataset of 1210 tweets, and applied the coding items. This process led to the elimination of irrelevant items and the inclusion of frequently used sub-items to assess HBM constructs and source attributes. For example, “social isolation/mental health issues” was added for perceived severity, and “vaccination passport/mandatory vaccine requirement” was included for perceived barriers. Ultimately, the coding scheme consisted of 35 items: 6 for susceptibility, 6 for severity, 9 for benefits, 11 for barriers, and 3 for self-efficacy. The final codebook is presented in Table 2.

### 3.3. Coding Procedure

To achieve a robust level of agreement, the coders participated in detailed training sessions where they learned the coding framework and practiced on a separate set of tweets not included in the main analysis (Krippendorff, 2018). Any differences were discussed until a consensus was reached, ensuring consistent coding. This preparation fostered a unified understanding of the coding categories and reduced potential ambiguities. Following training, each coder independently coded the entire dataset. Intercoder reliability was measured using Cohen’s kappa due to the categorical nature of the variables, yielding an average kappa of 0.94 (ranging from 0.8 to 1), indicating strong agreement. Table 2 presents the intercoder reliability coefficients between two coders for each coding item.

### 3.4. Model Specification

In this study, the Many-Facet Rasch Model (MFRM) was employed to examine the quality of reliability and validity assessments (Linacre, 1991). The MFRM allows for the examination of multiple facets simultaneously, providing a nuanced understanding of the data. FACETS 4.1 software was utilized for the analysis.

The MFRM is mathematically represented as follows:

$$ln\left[\frac{P_{jni,k}}{P_{jni,\;(k-1)}}\right]=\theta_{j}-\delta_{i}-\tau_{k}$$

where

- *P_jni_*_,*k*_ is the probability of post *j* receiving a rating *k* coded by *n* on item *i*;
- *P_jni_*_,(*k*−1)_ is the probability of post *j* receiving a rating *k* − 1 coded by *n* on item *i*;
- $\theta_{j}$ is the clarity measure for post *j*;
- $\delta_{i}$ is the difficulty of endorsing item *i*;
- $\tau_{k}$ is the difficulty of endorsing category *k* relative to *k* − 1.

### 3.5. Data Analysis

The log-odds ratio (logits) obtained from the MFRM represents a linear combination of latent measures across different facets, allowing for the creation of additive interval scales. Higher logit values indicate greater clarity for raters, while higher values for items and response categories signify greater difficulty in rating. Data analysis involved computing logit values for each facet, which were then visually represented using a Wright map. This map provided an empirical display of clarity scores and item difficulties for each HBM construct. Additionally, the reliability of separation indices were calculated for items, posts, raters, and Infit and Outfit Mean Square (MnSq) statistics. These indices indicate the reproducibility of the scale with different but equivalent samples. Infit and Outfit MnSq statistics were also computed to evaluate how well each item and person fit the latent scale, supporting the model’s internal structure validity.

## 4. Results

### 4.1. Descriptive Statistics of HBM Constructs

Table 3 displays the summary statistics of the observed and Rasch scores for each of the HBM constructs: (1) severity, (2) susceptibility, (3) benefits, (4) barriers, and (5) self-efficacy. The observed mean scores for the items ranged from 0.01 to 0.08, with the lowest being found for susceptibility (*SD* = 0.01) and the highest being found for barriers (*SD* = 0.09). The Rasch measure means varied from 0 to 0.53, where severity (*SD* = 1.44), benefits (*SD* = 1.64), barriers (*SD* = 1.63), and self-efficacy (*SD* = 1.02) all had a mean of 0, and susceptibility had the highest mean of 0.53 (*SD* = 1.77). In terms of Infit MnSq, the scores ranged from 0.91 to 1.01, with susceptibility presenting the lowest at 0.91 (*SD* = 0.4) and severity (*SD* = 0.21) and self-efficacy (*SD* = 0.19) having the highest at 1.01. The Outfit MnSq scores ranged from 0.88 to 1.5, with susceptibility having the lowest mean of 0.88 (*SD* = 0.86) and benefits the highest mean of 1.5 (*SD* = 1.18).

For tweets, the observed mean scores ranged from 0.01, with susceptibility again having the lowest (*SD* = 0.04), to 0.08, with barriers having the highest (*SD* = 0.06). The Rasch measure means were between −4.02 and −3.05, with susceptibility having the lowest mean of −4.02 (*SD* = 0.43) and self-efficacy the highest at −3.05 (*SD* = 0.73). The Infit MnSq scores ranged from 0.98 to 1.00, with severity at the lowest end (*SD* = 0.4) and susceptibility (*SD* = 0.41), benefits (*SD* = 0.29), and barriers (*SD* = 0.32) at the highest end. The Outfit MnSq scores varied from 0.88 to 1.2, with susceptibility having the lowest mean of 0.88 (*SD* = 0.86) and severity the highest at 1.2 (*SD* = 1.67).

For raters, the observed mean scores ranged from 0.01 to 0.08, with susceptibility having the lowest (*SD* = 0.00) and barriers the highest (*SD* = 0.01). The Rasch measure means of all five constructs were 0. The Infit MnSq scores ranged from 0.95 to 1.02, with severity having the lowest mean (*SD* = 0.01) and susceptibility the highest (*SD* = 0.01). Lastly, the Outfit MnSq scores ranged from 0.88 to 1.5, with susceptibility at the lowest end (*SD* = 0.22) and benefits at the highest end (*SD* = 0.72).

### 4.2. Reliability

**Figure 1:**
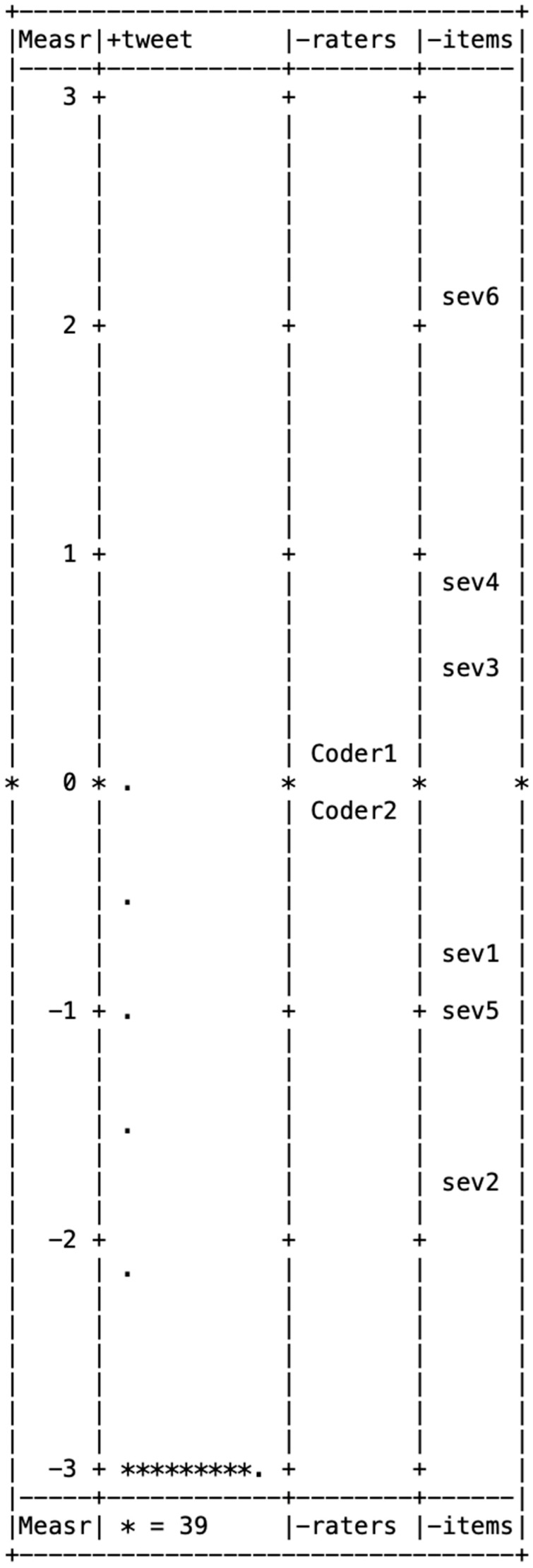
Wright map for severity.

**Figure 2:**
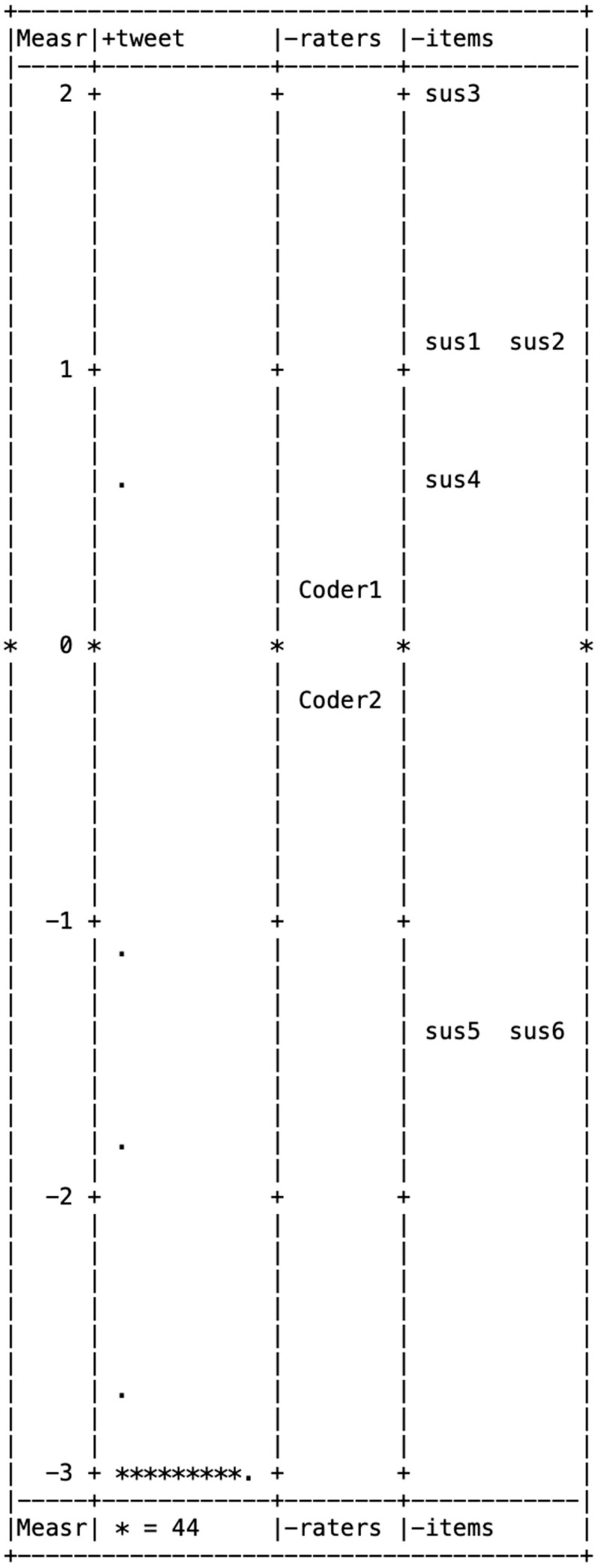
Wright map for susceptibility.

**Figure 3:**
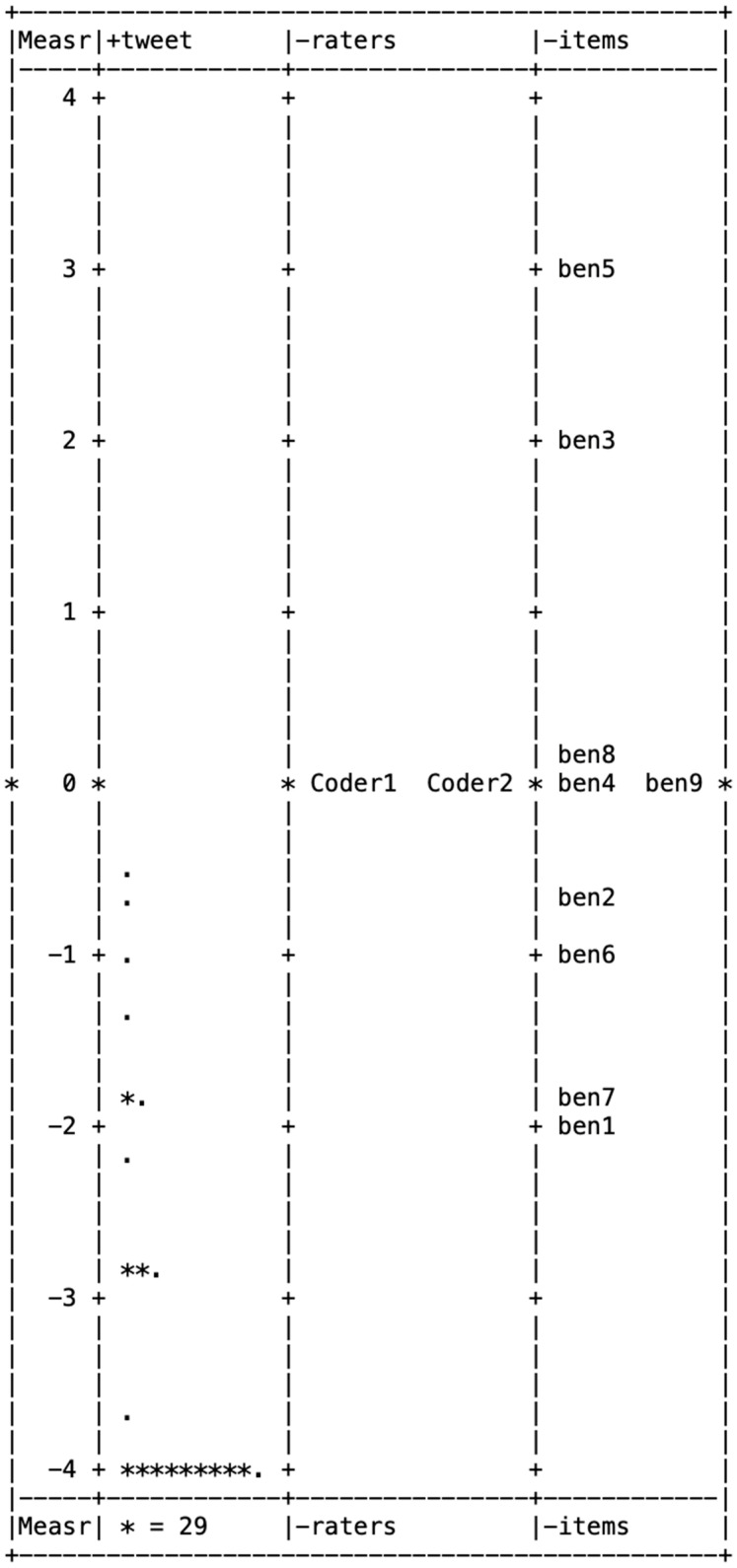
Wright map for benefits.

**Figure 4:**
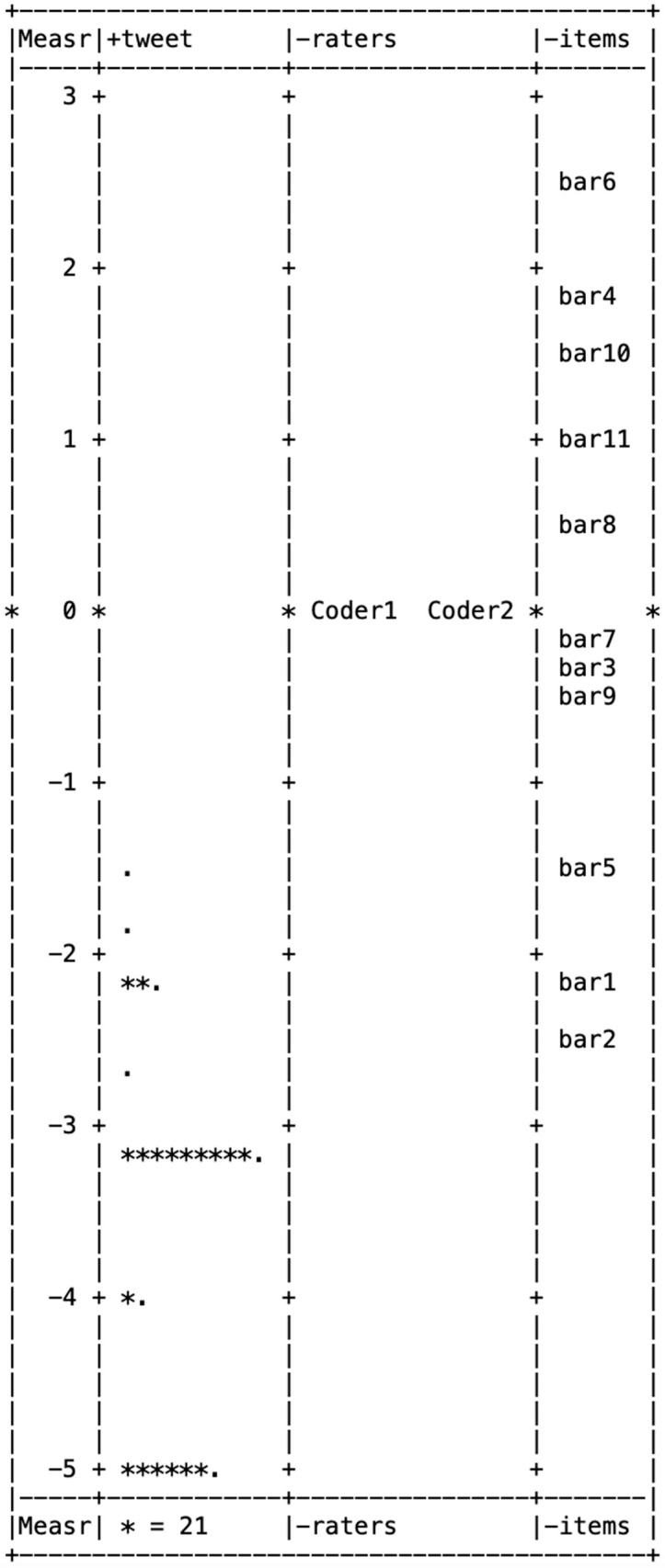
Wright map for barriers.

**Figure 5:**
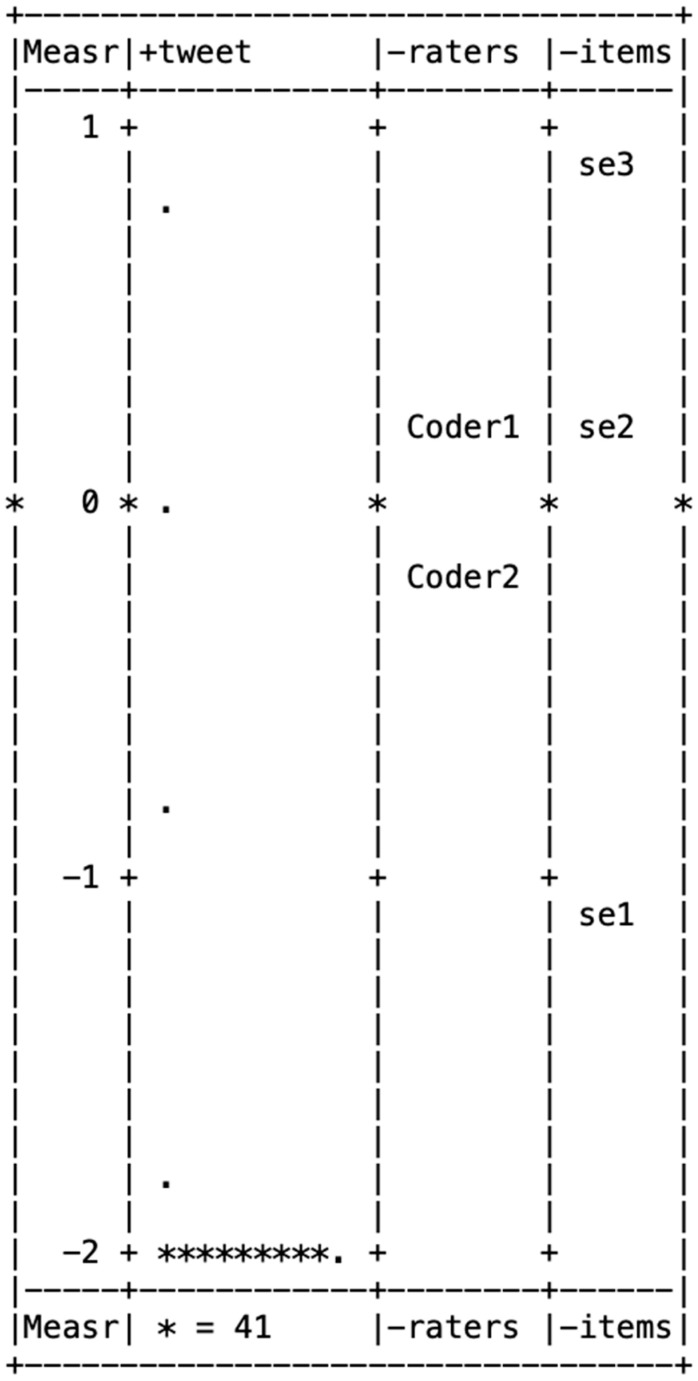
Wright map for self-efficacy. Note. In Figure 1, Figure 2, Figure 3, Figure 4 and Figure 5, * (Asterisk) indicates the presence of tweets at a specific measurement level, with the number of asterisks representing the count of tweets at that level. + (Plus sign) marks the measurement scale levels, dividing different points on the logit scale. . (dot) serves as a placeholder, indicating that no tweets were observed at that specific measurement level.

Figure 1, Figure 2, Figure 3, Figure 4 and Figure 5 illustrate the Wright maps, providing an empirical visualization of the scales for severity (Figure 1), susceptibility (Figure 2), benefits (Figure 3), barriers (Figure 4), and self-efficacy (Figure 5). Each figure consists of four columns: The first shows the Rasch scores on a logit scale, followed by the locations of tweets (column 2), raters (column 3), and items (column 4) based on their Rasch scores. The second column, which represents the distribution of tweets, can be understood as analogous to a vertical histogram, where the density of tweets at different Rasch score levels is visually depicted. The final column displays the threshold estimates of response categories on a Likert scale. All five figures are skewed to the right. This indicates that most tweets promoting COVID-19 vaccines did not emphasize each of the five HBM constructs as a strategy.

Among the six items in the severity item bank, three items—social isolation/mental health issues (item 6), family/friends dying (item 4), and COVID-19 has serious aftereffects (item 3)—were above the mean of 0, showing these aspects were less frequently highlighted in tweets about the COVID-19 vaccine. Of the six items in the susceptibility item bank, four—healthcare workers (item 3), elderly people (item 1), disadvantaged groups (item 2), and pregnant women (item 4)—had scores above the mean of 0, indicating these groups were less commonly referenced in tweets promoting the COVID-19 vaccine.

From the nine items in the benefits item bank, three—relief from worrying (item 5), feeling protected from COVID-19 infection (item 3), and saving medical resources (item 8)—were above the mean of 0, pointing out that these benefits were not commonly highlighted in vaccine promotion tweets. Out of the 11 items in the barriers item bank, 5—cannot accept injection (item 6), inconvenience of getting vaccinated (item 4), lack of family/friend support/social norm (item 10), misinformation/disinformation (item 11), and perceived rush (item 8)—had scores above the mean of 0, indicating these barriers were less frequently addressed in tweets about the COVID-19 vaccine.

Among the three items in the self-efficacy item bank, two—having the ability to deal with side effects (item 3) and getting vaccinated being free (item 2)—scored above the mean of 0, indicating these self-efficacy aspects were less frequently highlighted in vaccine promotion tweets.

### 4.3. Dimensionality

The Many-Facet Rating Scale Model (MFRM) analysis revealed that a single underlying factor accounted for the variances in each of the HBM constructs, as follows: severity (31.27% of variance across six items), susceptibility (23.69% of variance across six items), benefits (24.10% of variance across nine items), barriers (20.58% of variance across eleven items), and self-efficacy (20.18% of variance across three items).

### 4.4. Validity

Table 4 shows that the Infit and Outfit MnSq for severity items 2, 5, 1, 3, and 4 fell into a fit category of A, indicating a good fit to the tweets promoting COVID-19 vaccines. However, item 6, while fitting Infit MnSq category A, was unproductive based on its Outfit MnSq. The use of severity across the 449 tweets differed significantly, with *χ*^2^ (448) = 593.0 and *p* < 0.001, with 374 tweets not mentioning the severity of COVID-19. Tweet 447 had the highest severity score.

Table 5 highlights that the Infit and Outfit MnSq for susceptibility items 5 and 6 fell into a fit category of A, indicating a good fit. Items 4, 1, and 2, although fitting Infit MnSq category A, were less productive or unproductive in the Outfit MnSq. Item 3 fell into category D for both measures, marking it as unproductive. The use of susceptibility across the 449 tweets was not significantly different, with *χ*^2^ (448) = 108.6 and *p* = 1, with 434 tweets not mentioning the susceptibility of COVID-19.

Table 6 reveals that benefits items 1, 7, 6, 2, 4, 8, and 3 fit category A for both the Infit and Outfit MnSq, indicating a good fit to the tweets. Items 9 and 5, while fitting category A in the Infit MnSq, were less productive or unproductive in the Outfit MnSq. The use of benefits items across the 449 tweets differed significantly, with *χ*^2^ (448) = 684.8 and *p* < 0.001, with 288 tweets not mentioning the benefits of COVID-19 vaccination. Tweet 215 had the highest benefits score.

Table 7 shows that barriers items 2, 1, 5, 9, 3, 7, 8, 11, 10, and 4 fit category A for both the Infit and Outfit MnSq, indicating a good fit. Item 6, although fitting category A in the Infit MnSq, was less productive in the Outfit MnSq. The use of barriers items across the 449 tweets was not significantly different, with *χ*^2^ (448) = 387.1 and *p* = 0.98, with 139 tweets not mentioning the barriers to COVID-19 vaccination.

Table 8 demonstrates that all self-efficacy items fit category A for both the Infit and Outfit MnSq, indicating a good fit. The use of self-efficacy items across the 449 tweets was not significantly different, with *χ*^2^ (448) = 214 and *p* = 1.00, with 408 tweets not mentioning self-efficacy in relation to COVID-19 vaccination.

## 5. Discussion

In this research, Rasch modeling was used to determine the reliability and validity of severity, susceptibility, benefits, barriers, and self-efficacy items in evaluating social media posts in terms of their reliance on each HBM construct.

### 5.1. Reliability of HBM Items in Social Media Posts (RQ1)

In terms of reliability, the results indicate that severity, benefits, and barriers are the most consistently represented constructs in social media posts, whereas susceptibility and self-efficacy appear less frequently, limiting their overall reliability as indicators of the HBM in this context.

For severity, most items from the original HBM scale—such as “Item 2: COVID-19 can be fatal” and “Item 3: COVID-19 has serious aftereffects”—align well with the construct. However, “Item 6: Social isolation/mental health issues” demonstrates unexpected response variance, reducing its reliability. This suggests that while severity is a prominent factor in health messaging on social media, specific items may require refinement to improve measurement consistency.

Susceptibility, in contrast, is minimally represented in social media discussions, weakening its reliability. Items referring to vulnerable populations, such as “children” and “unvaccinated people”, perform relatively well, but others, such as “pregnant women” and “elderly people”, exhibit inconsistencies. This suggests that the construct of susceptibility is underutilized in vaccine-related posts, potentially limiting its effectiveness in influencing health behavior. Given that perceived susceptibility plays a key role in shaping health behaviors, this underrepresentation may limit the effectiveness of vaccine promotion efforts.

For benefits, the majority of the items align well with the construct, particularly statements emphasizing a reduced infection risk and restored social life. However, items such as “Item 5: Relief from worrying” display inconsistent responses, indicating a need for refinement. The strong presence of benefits-related messaging highlights the role of positive reinforcement in social media-based health campaigns, emphasizing that vaccination protects individuals and their communities.

Barrier items also effectively reflect the construct in tweets, except for “Item 6: Cannot accept injection”, which is rarely mentioned and shows unexpected response variance. Compared to other HBM constructs, 70% of the tweets are trying to target the barriers to getting the COVID-19 vaccine. This indicates that content creators recognize the importance of addressing barriers as a critical factor in persuading people to change their health behaviors (Laranjo et al., 2015).

Lastly, self-efficacy is similar to susceptibility, with only 41 tweets applying this strategy in their social media posts promoting the COVID-19 vaccine. According to previous research, perceived self-efficacy is a crucial element for people to adopt health behaviors (Leganger et al., 2000). Social media content creators need to emphasize the accessibility of the COVID-19 vaccine to influence people’s health behaviors effectively. In conclusion, all these findings suggest that while some items of the HBM constructs are effective, there is room to improve how these constructs are communicated in social media posts to enhance vaccine promotion efforts.

### 5.2. Validity of HBM Items in Social Media Posts (RQ2)

The findings suggest that while most HBM items effectively capture their intended constructs, their application in social media posts is sometimes inconsistent. Dimensionality analysis indicates that each construct captures distinct patterns, but the presence of unexplained variance suggests that other factors—beyond the HBM—may also shape social media vaccine messaging.

For severity, benefits, and barriers, items generally exhibit strong validity, aligning well with expectations from the Rasch model. The widespread use of severity-related messaging, particularly content emphasizing COVID-19’s fatality and transmission, suggests that social media campaigns prioritize risk-based persuasion strategies. Similarly, the benefits construct is well represented, particularly in relation to personal and community protection, reinforcing the idea that vaccine promotion campaigns often leverage messages about collective well-being. Barriers also demonstrate strong validity, but the lack of variation in how these messages are framed suggests that social media campaigns may benefit from more diverse approaches to addressing vaccine hesitancy.

Conversely, susceptibility and self-efficacy display weaker validity. The low representation of susceptibility-related content, despite its theoretical importance in the HBM, suggests that this construct may not be an effective predictor of vaccine uptake in the social media context. Likewise, the limited presence of self-efficacy-related messages indicates that social media campaigns rarely focus on individuals’ ability to overcome logistical or psychological barriers to vaccination. Given that prior research emphasizes the importance of self-efficacy in behavior change (Leganger et al., 2000), this gap suggests an area for improvement in social media-based health communication strategies.

### 5.3. Implications

This study is the first attempt to explore the psychometric properties of HBM measures evaluating social media posts promoting COVID-19 vaccination in terms of its use of HBM constructs using Rasch Measurement Theory (RMT). By employing RMT, this study provides a rigorous assessment of validity, reliability, and fairness, offering deeper insights into how health behavior promotion strategies function within social media contexts. The findings contribute to both theoretical and practical understandings of health communication in several important ways.

Firstly, this study advances the psychometric evaluation of HBM constructs in digital health messaging. Prior research has mainly concentrated on the interrater reliability of these constructs, neglecting the examination of item reliability and validity. This study provides guidelines for using HBM-related measures in social media by establishing comprehensive psychometric properties, especially when applied in social media contexts. We believe that our analysis demonstrates that some HBM constructs—severity, benefits, and barriers—are measured with greater precision, enhancing their applicability in future social media research, while suggesting the need to improve susceptibility and self-efficacy items before use in social media health messaging.

Second, the findings demonstrate the applicability of HBM measures beyond traditional surveys, highlighting how these constructs vary across social media posts. Notably, susceptibility and self-efficacy are underrepresented in vaccine-related posts, suggesting a theoretical and practical gap in their application to digital health communication that needs attention in future work. Addressing this gap could enhance the effectiveness of health messaging.

Additionally, this study offers valuable insights into the validity of specific items within each construct. The analysis shows that some items are considered less productive or unproductive based on their Outfit MnSq, which means that they exhibit unexpected variance in responses, reducing their effectiveness in evaluating the construct. For example, item 5 “Relief from worrying” in the benefits construct shows inconsistent responses, indicating that it might need refinement to improve its utility in accurately measuring benefits. This detailed understanding of item-level validity can guide public health officials and content creators in crafting more precise and effective health messages.

Furthermore, by refining the measurement of HBM constructs for social media posts, this study enhances their applicability in research models. With improved measurement, HBM constructs can more effectively serve as latent variables in causal analyses, allowing researchers to better examine the relationships between health beliefs and behavioral intentions in social media contexts.

Practically, these findings provide valuable insights for public health officials and social media content creators on the importance of emphasizing certain HBM constructs, such as self-efficacy and susceptibility, to create more persuasive health messages. Tailoring messages to highlight these elements could improve engagement and persuasiveness. Policymakers and campaign designers can also use these insights to develop more comprehensive health campaigns that incorporate multiple HBM constructs. Additionally, this study underscores the need for professional training programs to help social media managers and health communicators apply HBM principles effectively in digital health messaging.

### 5.4. Limitations and Future Research

This study has several limitations that should be addressed in future research. First, it does not assess whether HBM constructs function fairly across different social media posts and audience demographics. Future research should investigate measurement invariance to ensure broad applicability across various contexts. Additionally, this study focused on the psychometric properties of the HBM constructs in the context of COVID-19 vaccine promotion, which may limit the generalizability of the findings to other health behaviors or different types of health crises.

In addition, the dataset includes only the top 1210 tweets based on engagement, with 449 vaccine-related tweets forming the final sample. This selection may overlook perspectives from less prominent social media content and non-English-speaking communities. Additionally, since our study relies solely on data from the CoVaxxy dataset, which consists of Twitter posts, it does not capture perspectives from other social media platforms. Future research should consider incorporating data from multiple platforms to provide a more comprehensive understanding of how these constructs manifest across different social media environments.

Finally, while this study highlighted the variability in the emphasis on different HBM constructs and specific items across social media posts, it did not explore the reasons behind this variability. Future studies should investigate the factors that influence content creators’ decisions to emphasize certain HBM constructs over others. Understanding these factors can help in designing more effective health communication strategies.

## 6. Conclusions

In conclusion, this study represents a pioneering effort to evaluate the psychometric properties of Health Belief Model (HBM) constructs in the context of social media posts promoting COVID-19 vaccination using Rasch Measurement Theory. By examining the reliability and validity of these constructs, this research provides valuable insights into the varying emphasis on HBM constructs across tweets and highlights the complexity of health communication on social media platforms. While this study reveals the underutilization of certain constructs and the less effective or unproductive items of each construct, it underscores the importance of incorporating these elements to enhance message effectiveness. This study paves the way for future applications of HBM constructs in social media analyses. Additionally, it demonstrates the HBM’s applicability beyond traditional surveys, revealing variations in construct emphasis and highlighting the need to refine underutilized constructs and items for more effective health communication.

## Figures and Tables

**Table 1:** Fit categories for interpreting Infit and Outfit Mean Square Errors.

Mean Square Residual (MnSq)	Interpretation	Fit Category
0.5 < MnSq < 1.5	Productive for measurement	A
MnSq < 0.5	Less productive for measurement, but not distorting of measures	B
1.5 < MnSq < 2.0	Unproductive for measurement, but not distorting of measures	C
2.0 < MnSq	Unproductive for measurement, distorting of measures	D

**Table 2:** Coding sheet, intercoder reliability, and descriptive statistics.

Variables	Sub-Items	Number of Presences	Percent (%) of Presences	Cohen’s Kappa
Severity	Item 1: Death rate is high	23	5.12	0.97
	Item 2: COVID-19 can be fatal	35	7.80	0.93
	Item 3: COVID-19 has serious aftereffects	11	2.45	1.00
	Item 4: Family/friends dying	7	1.56	0.97
	Item 5: Widespread transmission	30	6.68	0.91
	Item 6: Social isolation/mental health issues	2	0.45	0.89
Susceptibility	Item 1: Elderly people	1	0.22	1.00
	Item 2: Disadvantaged groups	1	0.22	1.00
	Item 3: Healthcare workers	0	0.00	N/A
	Item 4: Pregnant women	1	0.22	0.80
	Item 5: Children	5	1.11	0.91
	Item 6: Unvaccinated people	6	1.34	0.91
Benefits	Item 1: Reduce the chance of infection	67	14.92	0.99
	Item 2: Decrease the severity and the chance of having complications	28	6.24	0.99
	Item 3: Feel protected from COVID-19 infection	3	0.67	1.00
	Item 4: Restore a normal social life	15	3.34	0.95
	Item 5: Relief from worrying	0	0.00	N/A
	Item 6: Protect family and friends, and others	37	8.24	0.99
	Item 7: Transmission reduction/end the pandemic	60	13.36	0.97
	Item 8: Save medical resources	15	3.34	0.98
	Item 9: Works for variants	17	3.79	0.95
Barriers	Item 1: Efficacy	90	20.04	0.94
	Item 2: Safety	121	26.95	0.97
	Item 3: Side effects	20	4.45	0.90
	Item 4: Inconvenience of getting vaccinated	3	0.67	1.00
	Item 5: Conspiracy theory	59	13.14	0.95
	Item 6: Cannot accept injection	1	0.22	0.80
	Item 7: Lack of knowledge/data	19	4.23	0.94
	Item 8: Rushed	10	2.23	0.90
	Item 9: Vaccination passport/mandatory vaccine requirement	31	6.90	0.92
	Item 10: Family/friends do not support/social norm	4	0.89	1.00
	Item 11: Misinformation/disinformation	6	1.34	0.92
Self-efficacy	Item 1: Getting vaccinated is easy	20	4.45	0.93
	Item 2: Getting vaccinated is free	10	2.23	0.95
	Item 3: Have ability to deal with side effects	6	1.34	0.92

*Note.* N/A: no data was available to calculate the intercoder reliability.

**Table 3:** Descriptive statistics.

Facets		Severity	Susceptibility	Benefits	Barriers	Self-Efficacy
Items						
Observed Scores	*M*	0.04	0.01	0.06	0.08	0.03
	*SD*	0.03	0.01	0.05	0.09	0.02
Rasch Measures	*M*	0	0.53	0	0	0
	*SD*	1.44	1.77	1.64	1.63	1.02
Infit MnSq	*M*	1.01	0.91	1	1	1.01
	*SD*	0.21	0.4	0.11	0.04	0.19
Outfit MnSq	*M*	1.3	0.88	1.5	0.98	1.02
	*SD*	0.69	0.71	1.18	0.25	0.25
Tweets						
Observed Scores	*M*	0.04	0.01	0.06	0.08	0.03
	*SD*	0.11	0.04	0.1	0.06	0.1
Rasch Measures	*M*	−3.85	−4.02	−3.95	−3.69	−3.05
	*SD*	1.08	0.43	1.31	1.17	0.73
Infit MnSq	*M*	0.98	1	1	1	0.99
	*SD*	0.4	0.41	0.29	0.32	0.58
Outfit MnSq	*M*	1.2	0.88	1.08	0.92	1.02
	*SD*	1.67	0.86	1.28	1.41	0.78
Raters						
Observed Scores	*M*	0.04	0.01	0.06	0.08	0.03
	*SD*	0	0	0	0.01	0.01
Rasch Measures	*M*	0	0	0	0	0
	*SD*	0.12	0.23	0.06	0.1	0.32
Infit MnSq	*M*	0.95	1.02	0.96	1	0.99
	*SD*	0.01	0.01	0.01	0.01	0.05
Outfit MnSq	*M*	1.3	0.88	1.5	0.98	1.02
	*SD*	0.21	0.22	0.72	0.04	0.12

**Table 4:** Summary of Rasch measures for severity items and precision of observational studies.

Item Number	Observed Score	Rasch Measures	SE	Infit		Outfit	
				MnSq	Category	MnSq	Category
2	0.09	−1.73	0.18	0.89	A	0.84	A
5	0.06	−1.04	0.18	1.05	A	1.09	A
1	0.05	−0.76	0.19	0.63	A	0.57	A
3	0.02	0.44	0.24	1.21	A	1.49	A
4	0.02	0.93	0.28	1.16	A	1.27	A
6	0.01	2.16	0.46	1.09	A	2.56	D

**Table 5:** Summary of Rasch measures for susceptibility items and precision of observational studies.

Item Number	Observed Score	Rasch Measures	SE	Infit		Outfit	
				MnSq	Category	MnSq	Category
5	0.01	−1.44	0.4	1.23	A	1.44	A
6	0.01	−1.44	0.4	0.94	A	0.9	A
4	0	0.62	0.65	1.37	A	1.71	C
1	0	1.12	0.77	0.52	A	0.16	B
2	0	1.12	0.77	0.52	A	0.16	B
3	0	3.21	1.84	Maximum	D	Maximum	D

**Table 6:** Summary of Rasch measures for benefits items and precision of observational studies.

Item Number	Observed Score	Rasch Measures	SE	Infit		Outfit	
				MnSq	Category	MnSq	Category
1	0.15	−1.95	0.12	0.85	A	0.83	A
7	0.14	−1.78	0.12	0.87	A	0.85	A
6	0.08	−0.99	0.14	0.98	A	1.01	A
2	0.06	−0.59	0.15	0.97	A	0.92	A
9	0.04	0.01	0.18	1.19	A	1.68	C
4	0.04	0.08	0.19	1.07	A	1.35	A
8	0.03	0.23	0.2	1.09	A	1.39	A
3	0.01	1.93	0.41	0.96	A	0.89	A
5	0	3.05	0.71	1.02	A	4.55	D

**Table 7:** Summary of Rasch measures for barriers items and precision of observational studies.

Item Number	Observed Score	Rasch Measures	SE	Infit		Outfit	
				MnSq	Category	MnSq	Category
2	0.28	−2.56	0.08	0.94	A	0.93	A
1	0.21	−2.1	0.09	1	A	1.03	A
5	0.14	−1.5	0.1	1.05	A	1.05	A
9	0.06	−0.5	0.14	1.06	A	1.27	A
3	0.05	−0.32	0.16	1.02	A	1.04	A
7	0.05	−0.25	0.16	0.96	A	0.89	A
8	0.02	0.47	0.22	0.96	A	0.78	A
11	0.02	0.94	0.27	1	A	0.93	A
10	0.01	1.51	0.36	1	A	0.97	A
4	0.01	1.81	0.41	1.01	A	1.41	A
6	0	2.51	0.58	0.99	A	0.46	B

**Table 8:** Summary of Rasch measures for self-efficacy items and precision of observational studies.

Item Number	Observed Score	Rasch Measures	SE	Infit		Outfit	
				MnSq	Category	MnSq	Category
1	0.05	−1.12	0.23	0.96	A	0.92	A
2	0.02	0.25	0.26	0.86	A	0.83	A
3	0.02	0.87	0.3	1.22	A	1.3	A

## Data Availability

The datasets used and/or analyzed during the current study are available from the corresponding author upon reasonable request.
